# The influence of Big Five personality traits on college students’ key competencies: the mediating effect of psychological capital

**DOI:** 10.3389/fpsyg.2023.1242557

**Published:** 2023-08-04

**Authors:** Anqi Hu, Xueyan Li, Hongfeng Song

**Affiliations:** School of Economics and Management, Beijing Forestry University, Beijing, China

**Keywords:** Big Five personality traits, psychological capital, key competencies, college students, higher education

## Abstract

**Background:**

In recent years, both society and employers have put forward higher requirements for the comprehensive quality of college students in the new era. Based on the conservation of resources theory and life-cycle approach, this study aimed to examine the relationship between the Big Five personality traits, the psychological capital, and the key competencies among college students and analyzed the mediating role of the psychological capital in this link.

**Methods:**

A total of 1,132 Chinese undergraduates (67.40% girls; 48.67% from key universities) participated. Participants completed self-report questionnaires that evaluated the five key characteristics of personality, psychological capital, and key competencies.

**Results:**

There were extremely significant university-type differences in key competencies of college students. And the mediating role of psychological capital in the link between Big Five personality traits and key competencies was validated according to PROCESS model 4. Psychological capital serves as a partial mediator in the relationships between neuroticism and critical thinking, openness and creativity, conscientiousness and creativity, openness and communication, conscientiousness and communication, extraversion and collaboration, as well as openness and collaboration. The proportion of mediating effects for the above models was 5.97, 10.89, 11.82, 12.24, 11.98, 12.39, and 22.72%, respectively.

**Discussion:**

The findings provide a better understanding of the key competencies of college students from the perspectives of the Big Five personality traits and psychological capital and suggest a greater emphasis to focusing on personality and improving psychological capital.

## 1. Introduction

The term “key competency” was first recognized and defined by the DeSeCo Project in 1997 as a pivotal concept in human resource management, education, and psychology, serving as a foundational framework for successful living and social functioning (Rychen and Salganik, 2003). The Chinese government, since the 18th CPC National Congress in 2012, has emphasized the cultivation of key competencies in college students, initiating curriculum reforms in 2014 and focusing on accelerating high-quality education system construction, as underscored by President Xi Jinping in 2022, to foster lifelong and social adaptability in students. “Higher education has become the new star ship in the policy fleet for governments which undertakes the mission of talent cultivation” (Olssen and Peters, 2005). To occupy the high ground of global competition in global technological revolution and industrial change, countries around the world are striving to cultivate talents that can meet the needs of society and have key competencies in the international arena (Lin, 2017).

A great deal of research has been conducted on key competencies of students, which focuses on its connotation, composition, and promotion path. And most of them were based on the background of civic education (Veugelers, 2011), putting forward countermeasure suggestions such as adjusting the talent cultivation system (Scheerens, 2011) and reforming the curriculum system (Banks, 2008). However, in the current field of pedagogy, scholars have focused on the primary and secondary education phase (Pepper, 2011; Ángel De-Juanas and Martín, 2016; Brečka and Valentová, 2017). Numerous studies have focused on the development of key competencies in specific areas or disciplines such as information skills (Martinez-Abad et al., 2016), English as a foreign language (Sun and Zhu, 2023), digital competences (Gorghiu et al., 2018; Zhao et al., 2021), etc. In summary, there is a gap in research on key competencies at the higher education level. In this context, an in-depth study of college student key competencies and a more targeted approach to the cultivation and promotion of key competencies are of great value and significance for adapting to the current higher education reform and increased demand for social talents, improving the employment situation of college students and improving the quality of college employment. of students.

Trait activation is the process by which “dormant” traits that are latent within individuals are awakened in appropriate contexts and manifest specific behaviors (Tett and Guterman, 2000). The specific behaviors resulting from trait activation are called “trait-expressive behavior” (TEB). As an innate personality trait, the Big Five personality traits (hereafter abbreviated as BFPT) is considered stable and unchangeable (McCrae and Costa, 1994; Caspi et al., 2005; Roberts et al., 2006), while psychological capital (commonly abbreviated in academia as PsyCap) is a positive psychological force that can be measured, nurtured, and developed through intervention (Luthans et al., 2008). Psychological study has shown that intellectual factors usually account for only 20% of the conditions that promote individual success, while nonintellectual factors account for 80% (Wang and Song, 2011). As a foundational personality trait, BFPT has an innate influence on key competencies. In the “traits to competencies” process, PsyCap may may be a trait-related cue (Tett and Burnett, 2003) that mediates the stimulation and expression of traits.

Hence, the objectives of the paper are tripartite. Firstly, explore possible differences in key competencies at the gender or institutional level; Secondly, examine the correlation between the dimensions of BFPT, PsyCap and college students’ key competencies; Thirdly, examine the mediating role of PsyCap between BFPT and key competencies. Therefore, this study has the following research questions: what is the relationship between BFPT and key competencies? How does PsyCap mediates the said relationship?

To achieve these objectives, this study is divided into six parts. The first part introduces the research background, research objectives, and questions. The second part theoretically constructs the process mechanism model of “BFPT→PsyCap→key competencies” and puts forward relevant research hypotheses. The third part introduces the data sources and research methods and explains the reliability of the measurement scale of related variables. The fourth section presents the results of difference test, correlation test and bootstrap mediation effect test to verify and test the hypotheses. The fourth part further discusses the results of the study. The fifth section gives the conclusions of this paper. The sixth section presents the points where the article could be improved.

## 2. Development of theory and hypotheses

### 2.1. Key competencies of college students

There has not come to a consensus on the concept of key competencies. We have summarized the representative concepts given by some organizations and scholars, as illustrated in Table 1. Given that the subjects of our study are Chinese university students, we would adhere to the definition and viewpoints of Lin (2016), especially the goal of cultivating key competencies “in order to meet the needs of their lifelong development and the development of society,” which is more in line with the current development of Chinese society and the background of the development of Chinese higher education.

**Table 1:** Definitions of key competencies.

Scholars/organizations	Definition
OECD (2005)	Key competencies consist of three core elements: (i) it brings benefits to society and individuals; (ii) it helps individuals meet important needs they face in a variety of contexts; and (iii) it is important to everyone
European Communities (2006)	The qualities that all individuals need to achieve personal fulfillment and development, become active citizens, to integrate into society, and be successful employed
UNESCO (2015)	The ability to explore, research, experiment and create, the ability to express and communicate verbally, and the higher-order skills involved in problem solving such as logical thinking, analysis, synthesis, deduction, reasoning, induction, and hypothesis
Rieckmann (2012)	Key competencies represent an extension of specific competencies, are transversal, multifunctional, and contextual, are essential for the achievement of social goals (e.g., sustainability), personal development, and require individuals to have strong reflective skills
Ministry of Education of the People’s Republic of China (2014)	Key competencies are more complex than skills and refer to the competencies that people should have and continue to develop in their learning and life today and in the future, including knowledge, skills, attitudes and values that can guide their actions
Lin (2016)	The essential character and key competencies that students gradually develop in the course of their education at the appropriate level to meet the needs of their lifelong development and the development of society. It is a combination of knowledge, skills, emotions, attitudes, and values that students need

Formed from the corresponding literature in the table.

A review of the literature reveals that research on key competencies defined the growth and development of students mainly from the perspective of skills and abilities, and that several different terminologies were used. For instance, basic skills (Ishikawa and Ryan, 2002), 21st century skills (Trilling and Fadel, 2009), soft skills (Abdul Karim et al., 2012), generic skill (Yin, 2018), employability skills (Suleman, 2018). The most widely known connotation framework is the framework for 21st century learning announced by the American Partnership for 21st Century Skills (Partnership for 21st Century Skills, 2011). Academics are increasingly recognizing that the literacies described in the P21 framework have become integral to the success of all students around the world, which contain critical thinking, creativity, communication, and collaboration (Keane et al., 2016). Moreover, we believe that these four competencies are highly consistent with Lin’s (2016) statement of “facilitating lifelong development and adapting to social development.” Therefore, we adopted the 4Cs key competencies framework proposed by P21 which contains critical thinking, creativity, communication, collaboration.

As educational reforms continue, college students are gradually showing less gender-specific differences in their key competencies, breaking down old gender stereotypes. Additionally, gender meta-analyzes have revealed that both sexes have similar levels in most psychological variables (Hyde, 2005). Based on this, our paper proposes the following.

*Hypothesis 1*. There are no significant gender differences in the key competencies of college students.

Standardized tests like the China College Entrance Examination or the SAT in the United States may play a role in the selection process, reflecting differences in the key competencies of college students across institution types. Apart from this, there may be different educational philosophies and teaching approaches in resources and opportunities (Bowen et al., 2005), Peer influence (Sacerdote, 2001), expectations and standards (Bowen et al., 2009). These differences in training models can lead to gaps in key competencies of students. Therefore, we proposed the following hypotheses:

*Hypothesis 2a (2b, 2c, 2d)*. There is a significant difference in critical thinking (creativity, communication, collaboration) for college students from different tiers of university.

### 2.2. BFPT and key competencies

Personality is a notable measurement of non-cognitive abilities, and psychologists have conceptualized personality traits primarily using self-esteem, internal locus of control, assertiveness, and anxiety (Feingold, 1994). After decades of scholarly efforts, the BFPT model was established and this has contributed to the dramatic growth of personality research since the 1980s (Digman, 1990). The Big Five framework enjoys considerable support and has become the most widely used and extensively researched model of personality (McCrae and Costa, 1994). The Big Five refers to the five dimensions that represent personality at the broadest level of abstraction; these five dimensions are typically labeled as extraversion, openness, agreeableness, neuroticism, and conscientiousness. According to the definition in Costa and McCrae’s NEO-PI-R test manual, which is the most commonly accepted definition nowadays, the brief explanation of each trait is as follows: (1) neuroticism pertains to emotional instability, marked by anxiety, hostility, depression, self-consciousness, impulsivity, and vulnerability. (2) Extraversion embodies sociability and outgoingness, characterized by warmth, gregariousness, assertiveness, activity, a penchant for excitement, and positive emotions. (3) Openness indicates receptivity to new experiences, ideas, and feelings, defined by imagination, aesthetic sensitivity, emotional depth, adventurousness, intellectual curiosity, and a propensity to challenge conventional wisdom. (4) Agreeableness measures interpersonal harmony and cooperation, characterized by trust, straightforwardness, altruism, compliance, modesty, and tenderness. (5) Conscientiousness represents organizational skills, responsibility, and thoroughness, indicated by competence, orderliness, dutifulness, a drive for achievement, self-discipline, and careful consideration. (Costa and McCrae, 2008).

Numerous academics have conducted research on the correlation between BFPT and key competencies, such as perceived stress (You et al., 2020), perception of competencies development and personal preferences (López-López et al., 2020), global competencies and achievement in learning English (Cao and Meng, 2020), cognitive competencies (Cerni et al., 2021). Previous meta-analysis has found that among BFPT, neuroticism was the only trait negatively correlated with personal competencies (Poropat, 2009), while studies have revealed that extraversion (Barrick et al., 2001), openness (Chamorro-Premuzic and Furnham, 2003), agreeableness (Avey et al., 2010) and conscientiousness (Judge et al., 2007) were positively related to key competencies in academic and work areas. Due to a lack of data, there is currently no existing research using Chinese university students as a sample to study the relationship between BFPT and key competencies. Consequently, we formulate the following hypothesis.

*Hypothesis 3*. There are significant correlations between BFPT and key competencies. More specifically, neuroticism is negatively correlated, while extraversion, openness, agreement, and conscience are positively correlated.

### 2.3. The mediating role of Psycap

BFPT factors are found to be substantially influenced by genetic factors, largely formed during early childhood, and remarkably stable during young adulthood (Caspi et al., 2005). Building on the foundational work of Seligman (2002) in positive psychology, the concept of PsyCap was conceived, which Luthans (2002) later incorporated into the realm of management studies. Separately, Seligman’s theory of learned optimism has provided strategies to promote optimism and thus improve PsyCap (Seligman, 2006). Luthans (2002) found that PsyCap is a positive psychological force that can positively motivate positive attitudes and behaviors, promote physical and mental growth, academic and employment development, and enhance one’s competitive advantage. As a positively-oriented, renewable, and non-scarce key resource, PsyCap denotes the evolving positive mental state of an individual (Luthans and Youssef, 2004). Its effective development and management significantly impact key competencies (Luthans et al., 2007). Notably, PsyCap can be invested in and developed through psychological capital interventions (PCI) to unlock individual potential (Luthans et al., 2007). High PsyCap people have enough key resources to clearly position and maximize the strengths of personality traits (Xing et al., 2023). According to conservation of resources theory (COR), high psychological capitalists can continuously create more psychological resources through the resource gain spiral effect (Hobfoll, 2002). In summary, the study of PsyCap has important and far-reaching value for a comprehensive and in-depth understanding of the positive forces in individual traits (Xiong and Ye, 2014), positioning it as a strategic asset for maintaining long-term talent competitiveness (Ren et al., 2013). PsyCap is measurable, developable and cultivable and consists of four core elements: optimism, resilience, self-efficacy, and hope. The Luthans approach to this dimension of PsyCap has gained wide acceptance and has been highly cited in the academic community. As defined by Luthans et al. (2007): (1) self-efficacy refers to the confidence in one’s skills to successfully tackle tasks and attain goals. It underlines an individual’s belief in their capability to manage and perform in diverse situations. (2) Optimism is the positive anticipation of future outcomes. It showcases an individual’s tendency to view success as a product of enduring, universal factors and setbacks as temporary, situational incidents. (3) Hope encompasses the persistent drive to fulfill aspirations and the adaptability to modify strategies in the face of challenges. It signifies both grit in pursuing goals and flexibility when faced with hurdles. (4) Resilience represents the capacity to recover from and thrive amidst adversity. It underscores the ability to bounce back from distressing experiences and leverage adversity to fuel personal growth and success.

BFPT and PsyCap are interrelated, but do not overlap (Hong et al., 2020). PsyCap provides a unique perspective beyond stable personality traits in predicting individual performance (Luthans et al., 2007). PsyCap, with its state-like essence, offers a distinctive malleability (Luthans and Youssef, 2004), it’s not only subject to change but can be actively cultivated and enhanced through targeted interventions (psychological capital interventions, PCI) (Luthans et al., 2007). Contrastingly, BFPT serve as robust markers of an individual’s innate character, with meta-analyses underscoring their pronounced stability (Roberts and DelVecchio, 2000), particularly during adulthood (McCrae and Costa, 1994). Thus, While PsyCap offers a fluid and evolvable pathway for individual growth, BFPT provide a relatively stable and enduring representation of an individual’s inherent disposition.

**Figure 1:**
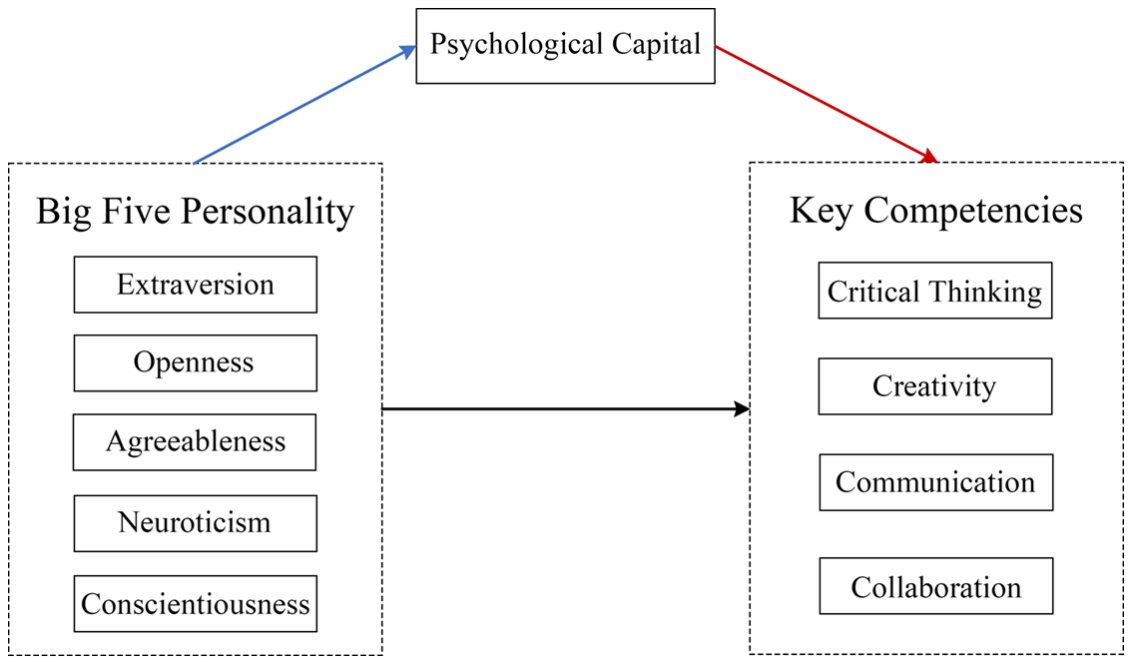
Diagram of the mediation research model.

The mediating role of PsyCap has been convinced between BTPT and subjective well-being (Luthans et al., 2007), academic performance (Luthans et al., 2019), career adaptability (Selma, 2022), investment performance (Akhtar and Das, 2020), which are representations of key competencies in career-oriented or learning-focused scenarios. Thus, we conjecture that PsyCap acts as a mediatorial bridge between BFPT and key competencies. Therefore, a theoretical model of “BTPT→PsyCap→key competencies” was developed (see Figure 1, below), and the hypotheses were proposed as follows:

*Hypothesis 4*. There are significant correlations between BFPT and PsyCap. Neuroticism is negatively correlated, while extraversion, openness, agreement, and conscientiousness are positively correlated.

*Hypothesis 5*. PsyCap mediates the relationship between BFPT and key competencies.

## 3. Materials and methods

### 3.1. Study design

This cross-sectional study was conducted in universities in China using convenience sampling. The questionnaires were distributed online and all participants were informed of the details of the study and could withdraw from participation at any time, either temporarily or permanently. Ethical approval was obtained from Beijing Forestry University.

### 3.2. Participants

A total of 1,253 undergraduate students participated in the study. After excluding invalid samples, we finally collected 1,132 valid questionnaires with an effective response rate of 90.34%. The exclusion of “invalid questionnaires” in this study was based on the following: Firstly, questionnaires that contain a multitude of unanswered questions or where key items are left blank were classified as invalid. Secondly, if the respondents chose the same option for all items, indicating a lack of engagement or careful consideration, the questionnaire was usually deemed invalid. Lastly, if the questionnaire was filled out in a time frame that is implausibly short, it may suggest rushed, non-thoughtful responses, rendering it invalid. The participants included 369 (32.60%) boys and 763 (67.40%) girls. Of the participants, 551 (48.67%) were from key universities^1^ and 581 (51.33%) were from general universities.^2^ Table 2 summarizes the distribution of valid questionnaires.

**Table 2:** Sample distribution statistics of the questionnaire.

Basic characteristics		Number of samples	Percentage
Gender	Male	369	32.60%
	Female	763	67.40%
Type of universities	Key universities	551	48.67%
	General universities	581	51.33%

### 3.3. Measures

At the beginning of this study, we made sure to convey the scholarly intention and the confidentiality assurance of the questionnaire. This was done with the objective of mitigating the responses’ apprehensions and thus collecting more authentic data. The main questionnaire contained two main parts: the first part was a background information, containing information on the type of universities, individual gender, etc. The second part includes the BFPT inventory, the PsyCap inventory and the college students’ key competencies inventory. The questionnaire responses were measured using a 5-point Likert scale (1 = very non-conformist, 5 = very conformist).

BFPT was measured using the Chinese version of the 10-item BFPT Inventory (TIPI-C), which was translated and developed by Li (2013) based on the 10-item BFPT Inventory (TIPI) developed by Gosling et al. (2003), which included five dimensions: (1) extraversion, (2) agreeableness, (3) consciousness, (4) neuroticism, (5) openness. TIPI-C was revealed to be reliable and valid to measure BFPT among Chinese university students, with a Cronbach’s alpha (*α*) of 0.863 in our study. The Cronbach’s alpha (*α*) coefficients of the five personality traits subscales reached 0.785, 0.709, 0.850, 0.820 and 0.700, respectively.

The PsyCap of the students was assessed using the positive PsyCap questionnaire (PPQ) developed by Zhang et al. (2010), which included four dimensions: hope, effectiveness, resilience, and optimism. Cronbach’s alpha (*α*) for this scale in this study was 0.874, with good reliability. This approach is widely accepted in existing research. Many researchers have used similar or identical methods to measure overall levels of PsyCap. It should be noted that in the follow-up study, PsyCap was expressed as a weighted average of four dimensions calculated using principal component analysis to indicate its overall level, this approach is widely accepted in existing research, and many researchers have used similar or identical methods to measure overall levels of PsyCap (Sui et al., 2012; Baron et al., 2016). Thus, the use of the weighted average of these four dimensions as a representation of the overall PsyCap is theoretically justified.

We selected four dimensions of key competencies for an in-depth study: critical thinking, creativity, communication, and collaboration. Given that there is no directly usable “key competencies assessment scale for college students” (with multiple dimensions), but only separate scales that distinguish between competency points, we aggregated the subscales of the four competency points to collectively measure the key competencies of college students. The four competencies were evaluated using the critical thinking inventory, creativity inventory, communication inventory and collaboration inventory developed by Gan et al. (2020), Kang et al. (2020), Ma et al. (2020) and Xu et al. (2020), respectively. The creators of the scale are very authoritative in the field of key competencies education for Chinese college students, so the scale has a high degree of recognition. The overall Cronbach’s alpha (*α*) for this scale in this study was 0.925, with good reliability. The Cronbach’s alpha (*α*) coefficients of the four competencies subscales reached 0.759, 0.942, 0.925, and 0.921, respectively. In addition, in order to test whether the collected data functioned according to the way these four sub-points are structured, a validation factor analysis was conducted using Mplus 8.0 for the scale items of the key competencies, which resulted in a fit index of (*χ*^2^/*df* = 2.963 < 3，CFI = 0.952 > 0.9, TLI = 0.945 > 0.9, RMSEA = 0.048 < 0.08) for the four-factor model, proving a high discriminant validity and a acceptable fit between critical thinking, creativity, communication, and collaboration.

### 3.4. Statistical analyses

Data were analyzed using SPSS 23.0 and PROCESS Model 4 (Hayes, 2013). The reliability of all the measurement instruments in this study was calculated using Cronbach’s alpha (*α*). Before analysis, normality, homoscedasticity, and linearity were examined and found to be supported. The correlation between each variable was derived from the Pearson bivariate product-moment correlation coefficient (*r*). Standard regression and the bootstrap method were used to test the mediation hypothesis. Compared to traditional stepwise testing (Baron and Kenny, 1986) and the Sobel method (Sobel, 1982), bootstrap is less demanding on the sample and more sensitive in determining the model. In this study, 5,000 bootstrap samples were used. BFPT served as a predictor, PsyCap as mediator, and key competencies of college students as the outcome variable. Age, sex, type of universities, and the other 4 dimensions of BFPT except for the independent variable *X* were set as covariates based on previous studies (González-Morales et al., 2012). Direct and indirect effects were calculated to determine the results of the mediation model. Confidence intervals (CI) that did not contain 0 were considered significant. Statistical significance was established at *p* < 0.05.

## 4. Results

### 4.1. Descriptive analysis and variance analysis of key competencies

#### 4.1.1. Descriptive analysis of key competencies of college students

Table 3 reports the results of the descriptive analysis. The results indicated that the overall level of key competencies among Chinese college students was high, with a mean score of 4.543 (maximum score of 5). Moreover, the overall levels of all four of these subdimensions were relatively high. Dimension 3, communication, had the highest level (4.567 ± 0.086), followed by dimension 4, collaboration (4.555 ± 0.089) and dimension 1, critical thinking (4.542 ± 0.101), and lower levels for dimension 2, creativity (4.501 ± 0.095) in comparison. The consistency of these scores was notable, with all means and medians quite similar. This suggested that students tend to be balanced in their competencies, without extreme strengths or weaknesses in these four areas.

**Table 3:** Results of the descriptive analysis of key competencies of college students.

Dimension	Mean value	Standard deviation	Median value
Critical Thinking	4.542	0.101	4.536
Creativity	4.501	0.095	4.492
Communication	4.567	0.086	4.559
Collaboration	4.555	0.089	4.547

#### 4.1.2. Variance analysis of key competencies in gender and university type

Independent sample *t*-test analysis was performed with key competencies as the dependent variable and gender and type of university as the independent variables. Table 4 reports the results of the gender difference test. It was found that both boys and girls showed consistency in all four dimensions of the key competencies, and there were no differences. Therefore, Hypothesis 1 was supported.

**Table 4:** *t*-test analysis of gender and university type differences in core competencies.

Dimension	Gender (*M* ± SD)		*T*	Type of universities (*M* ± SD)		*T*
	Female (*N* = 763)	Male (*N* = 369)		Key univ. (*N* = 551)	General univ. (*N* = 581)	
Critical Thinking	4.54 ± 0.11	4.54 ± 0.09	−0.056	4.55 ± 0.67	4.53 ± 0.14	−2.458^*^
Creativity	4.50 ± 0.10	4.50 ± 0.07	−0.485	4.51 ± 0.81	4.49 ± 0.12	−2.225^*^
Communication	4.57 ± 0.08	4.57 ± 0.07	−0.161	4.57 ± 0.68	4.56 ± 0.11	−1.552
Collaboration	4.55 ± 0.10	4.55 ± 0.07	0.208	4.56 ± 0.70	4.55 ± 0.11	−1.747

^*^Means 0.05, ^**^means 0.01, ^***^means 0.001. The same as below.

There were significant differences in key competencies by type of university, as indicated in Table 4. There were significant differences in the key competencies of 2 aspects of critical thinking and creativity (Sig <0.05). In terms of critical thinking, the scores of the students from key universities (4.55 ± 0.67) were significantly higher than those of the students from general universities (4.53 ± 0.14), while in terms of the creativity, the scores of students from key universities (4.51 ± 0.81) were significantly higher than those of the students from general universities (4.49 ± 0.12). These differences may be related to the way student thinking is guided or nurtured in different schools. However, in terms of communication and collaboration, there was no significant difference between major and general universities, which did not have sufficient.

### 4.2. Correlation analysis among BFPT, Psycap, and key competencies

The results of the correlation between BFPT and key competencies of college students are shown in Table 5. There was a negative correlation between neuroticism and the four subdimensions of key competencies and the total score, showing that the higher the score of neuroticism, the lower the level of college students in all aspects. There were significant positive correlations between the dimensions of extraversion, openness, agreeability, conscientiousness, and the four subdimensions of key competencies and the total score, which revealed that the higher the score of these four dimensions, the higher the level of key competencies of college students in all aspects. The results therefore supported Hypothesis 3.

**Table 5:** Correlations between BFPT and key competencies, PsyCap and key competencies.

Dimension	Critical thinking	Creativity	Communication	Collaboration
Extraversion	0.073^**^	0.145^***^	0.143^***^	0.119^***^
Openness	0.139^***^	0.180^***^	0.211^***^	0.199^***^
Agreeableness	0.175^***^	0.193^***^	0.210^***^	0.192^***^
Neuroticism	−0.02	−0.070^**^	−0.082^***^	−0.061^**^
Conscientiousness	0.191^***^	0.231^***^	0.284^***^	0.278^***^
Optimism	0.273^***^	0.246^***^	0.246^***^	0.285^***^
Resilience	0.187^***^	0.159^***^	0.147^***^	0.218^***^
Self-efficacy	0.325^***^	0.306^***^	0.266^***^	0.326^***^
Hope	0.338^***^	0.307^***^	0.311^***^	0.324^***^

The results of the correlation between BFPT and PsyCap are shown in Table 6. The results demonstrated that there were significant negative correlations between neuroticism and the four dimensions of PsyCap: optimism, resilience, self-efficacy, and hope. There were significant positive correlations between the other four dimensions of BFPT and the four dimensions of PsyCap. Therefore, Hypothesis 4 was accepted.

**Table 6:** Correlations between BFPT and PsyCap.

Dimension	Extraversion	Openness	Agreeableness	Neuroticism	Conscientiousness
Optimism	0.216^***^	0.265^***^	0.229^***^	−0.131^***^	0.344^***^
Resilience	0.262^***^	0.290^***^	0.408^***^	−0.389^***^	0.371^***^
Self-efficacy	0.265^***^	0.291^***^	0.278^***^	−0.184^***^	0.361^***^
Hope	0.243^***^	0.319^***^	0.354^***^	−0.264^***^	0.404^***^

Table 5 reports the results of the correlation analysis between the four dimensions of PsyCap and the key competencies. There were significant positive correlations between the four dimensions of PsyCap and the four dimensions of key competencies and the total score, showing that the higher the score of PsyCap, the higher the level of key competencies.

### 4.3. Mediating effects of Psycap between BFPT and key competencies

The mediating effect of psycap in the relationship between BFPT and key competencies was examined according to the mediating effect procedure recommended by Wen and Ye (2014), controlling for the type of universities, gender, and the other four dimensions of BFPT except for the independent variable *X*. And model 4 of PROCESS (Hayes, 2013) was used to examine the possible mediating effect. Analysis was carried out using the nonparametric percentile bootstrap method (with a sampling size of 5,000). Confidence intervals (CI) that did not contain 0 were considered significant.

This section examined the relationship between the five dimensions of BFPT and the four dimensions of key competencies, using the weighted average of PsyCap as the mediating variable, with a total of seven significant mediation models resulting from the analysis.

To test the possible mediating effect of PsyCap between the five dimensions of BFPT and the four dimensions of key competencies, we constructed the following mediating effect model based on stepwise regression.


$$Y_{i}=\alpha_{0}+\alpha_{1}X_{j}+\gamma_{0}\text{Control}$$

$$M=\beta_{0}+\beta_{1}X_{j}+\gamma_{1}\text{Control}$$

$$Y_{i}=\delta_{0}+\delta_{1}X_{j}+\delta_{2}M+\gamma_{2}\text{Control}$$

In the formula: *Y* is key competencies, *X* is BFPT, *M* is PsyCap, and Control is the control variable (gender, type of university, and the other 4 dimensions of BFPT except for the independent variable *X*). *i* (=1, 2, 3, 4) indicates the 4 dimensions of key competencies (critical thinking, creativity. Communication and collaboration), and *j* (=1, 2, 3, 4, 5) marks the 5 dimensions of BFPT (extraversion, openness, acceptableness, neuroticism, and conscientiousness). *α*_0_, *β*_0_, and *δ*_0_ are constant terms.

#### 4.3.1. Principal component analysis of PsyCap

Using SPSS 23.0 statistical analysis software, a factor analysis was performed at the PsyCap level of Chinese university students. To eliminate possible adverse effects due to differences in magnitude, we have standardized the raw data.

The KMO was 0.768 (>0.7) and the Sig was less than 0.05, indicating that the analysis of the data supported the principal components. Meanwhile, a common factor was extracted according to the principle that the eigenvalue is greater than one, and the cumulative variance contribution was 73.370%. As a result, a common factor was extracted to reflect 73.370% of the variance of the original variable, as shown in Table 7.

**Table 7:** Total variance explained.

Ingredients	Initial eigenvalue			Extraction of sum of squares of loads		
	Total	Percentage variance	Cumulative percentage	Total	Percentage variance	Cumulative percentage
1	2.935	73.370	73.370	2.935	73.370	73.370
2	0.551	13.773	87.143			
3	0.305	7.617	94.760			
4	0.210	5.240	100.000			

This study employed the variance contribution of the principal components as weights. The normalization of the weighted average of the coefficients in the linear combination of each principal component for this index was executed. The weights of the indices were calculated using the principal component analysis method.

First, the coefficients of the linear combination were calculated using the formula.


$$w_{i}=\frac{F_{i}}{\mu }$$

In the above equation, $w_{i}$ is the coefficient in the linear combination corresponding to component 1 of the *i*th indicator, $F_{i}$ is the component matrix value corresponding to component 1 of the ith indicator, and μ is the square root of the eigenvalue of component 1; *i* = 1, 2, 3, 4. This results in a composite score model.


$$Y=w_{1}X_{1}+w_{2}X_{2}+w_{3}X_{3}+w_{4}X_{4}$$

This gave the formula for calculating the level of PsyCap.


$$\text{PsyCap}=0.4886\;X_{1}+0.4670\;X_{2}+0.5281\;X_{3}+0.5141\;X_{4}$$

#### 4.3.2. Mediated influence pathways test of critical thinking

In this section, the mediating paths of BFPT through PsyCap influencing critical thinking were tested, yielding a significant mediating model 1: neuroticism-PsyCap-critical thinking. The result indicates that neuroticism reduces performance in critical thinking partially by diminishing PsyCap.

**Figure 2:**
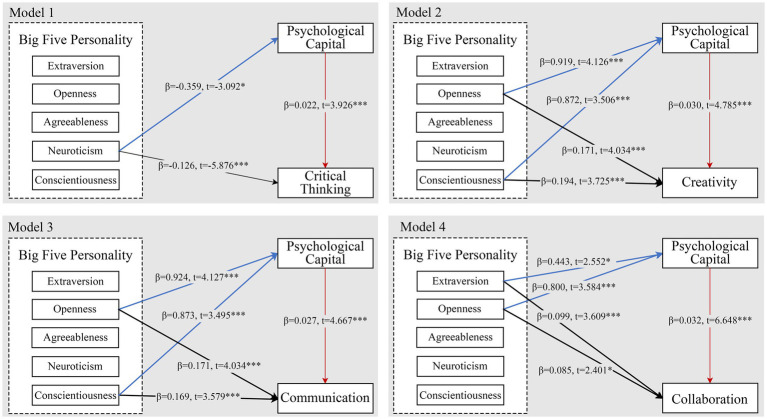
Mediating effect paths for models 1–7.

The results of the mediation analysis of model 1 are presented in Table 8 After controlling for the type of universities, gender, and the other 4 dimensions of BFPT, we first found that neuroticism negatively predicted critical thinking, *B* = −0.134, *p* < 0.001 (Eq. 1). Second, neuroticism negatively predicted PsyCap, *B* = −0.359, *p* < 0.1 (Eq. 2). Third, after putting both independent and mediating variables in the equation, neuroticism negatively predicted critical thinking, *B* = −0.126, *p* < 0.001, PsyCap positively predicted critical thinking, *B* = 0.022, *p* < 0.001 (Eq. 3). The bias-corrected bootstrapping mediation test indicated that the process by which neuroticism predicted critical thinking through PsyCap was significant, indirect effect = −0.008, 95% CI = (−0.016, −0.002), which are presented in the Table 9. The ratio of direct and mediated effects to the total effect was 94.03% and 5.97%, respectively, which was a partially mediated model. The mediated path diagram of model 1 is shown in Figure 2.

**Table 8:** Mediation model 1 test: neuroticism-PsyCap-critical thinking.

Predictors	Equation 1 (explicit variable: critical thinking)		Equation 2 (explicit variable: PsyCap)		Equation 3 (explicit variable: critical thinking)	
	*B*	*t*	*B*	*t*	*B*	*t*
PsyCap					0.022	3.926^***^
Neuroticism	−0.134	−6.227^***^	−0.359	−3.092*	−0.126	−5.876^***^
Gender	−0.050	−1.554	0.143	0.821	−0.050	−1.554
Type of universities	0.130	4.383^***^	0.285	1.766	0.130	4.383^***^
Extraversion	−0.070	−2.161^*^	0.353	2.019^*^	−0.070	−2.161^*^
Openness	0.021	0.516	0.922	4.107^***^	0.021	0.516
Agreeableness	0.136	3.608^***^	0.436	2.126^*^	0.136	3.608^***^
Conscientiousness	0.089	1.929	0.875	3.491^***^	0.089	1.929
*R* ^2^	0.163		0.238		0.175	
*F*	30.601		49.127		29.053	

The variables in the model are added to the regression equation using standardized variables. The same as below.

**Table 9:** Bia-corrected bootstrapping test in mediating effect of models 1–7.

Pathways	Effect	Boot SE	95% confidence interval		Percentage
			Boot LLCI	Boot ULCI	
Model 1: total effect	−0.134	0.021	−0.176	−0.092	
Neuroticism-PsyCap-critical thinking	−0.008	0.004	−0.016	−0.002	5.97%
Neuroticism-critical thinking	−0.126	0.021	−0.168	−0.084	94.03%
Model 2: total effect	0.257	0.047	0.165	0.349	
Openness-PsyCap-creativity	0.028	0.010	0.010	0.050	10.89%
Openness-creativity	0.229	0.047	0.138	0.321	89.11%
Model 3: total effect	0.220	0.052	0.118	0.323	
Conscientiousness-PsyCap-creativity	0.026	0.010	0.007	0.049	11.82%
Conscientiousness-creativity	0.194	0.052	0.092	0.296	88.18%
Model 4: Total effect	0.196	0.043	0.112	0.279	
Openness-PsyCap-communication	0.024	0.009	0.009	0.044	12.24%
Openness-communication	0.172	0.042	0.088	0.255	87.76%
Model 5: Total effect	0.192	0.047	0.099	0.286	
Conscientiousness-PsyCap-communication	0.023	0.009	0.007	0.043	11.98%
Conscientiousness-communication	0.169	0.047	0.077	0.262	88.02%
Model 6: total effect	0.113	0.028	0.058	0.168	
Extraversion-PsyCap-collaboration	0.014	0.007	0.001	0.028	12.39%
Extraversion-collaboration	0.099	0.027	0.045	0.153	87.61%
Model 7: total effect	0.110	0.036	0.040	0.181	
Openness-PsyCap-collaboration	0.025	0.010	0.006	0.047	22.72%
Extraversion-collaboration	0.085	0.035	0.016	0.155	77.27%

Boot SE, Boot LLCI and Boot ULCI refer to the standard error, lower bound and upper bound of the 95% confidence interval of the indirect effect estimated by the bia-corrected bootstrapping test, respectively; all values are rounded to retain two decimal places by rounding, as below.

#### 4.3.3. Mediated influence pathways test of creativity

In this section, the mediating paths of BFPT through PsyCap influencing creativity were tested, yielding 2 significant mediating models: the model 2 was openness-PsyCap-creativity, the model 3 was conscientiousness-PsyCap-creativity. The results indicate that openness and conscientiousness both improve performance in creativity partially by enhancing PsyCap.

The results of the mediation analysis of model 2 are presented in Table 10. After controlling for the type of universities, gender, and the other 4 dimensions of BFPT, we first found that openness positively predicted creativity, *B* = 0.257, *p* < 0.001 (Eq. 1). Second, openness positively predicted PsyCap, *B* = 0.919, *p* < 0.001 (Eq. 2). Third, after putting both the independent and the mediating variables in the equation, openness positively predicted creativity, *B* = 0.229, *p* < 0.001, PsyCap positively predicted creativity, *B* = 0.030, *p* < 0.001 (Eq. 3). The bias-corrected bootstrapping mediation test indicated that the process by which openness predicted creativity through PsyCap was significant, indirect effect = 0.028, 95% CI = (0.010, 0.050), which are presented in Table 9. The ratio of direct and mediated effects to the total effect was 89.11% and 10.89% respectively, which was a partially mediated model. The mediated path diagram of model 2 is shown in Figure 2.

**Table 10:** Mediation model 2 test: openness-PsyCap-creativity and model 3 test: conscientiousness-PsyCap-creativity.

Predictors	Equation 4 (explicit variable: creativity)		Equation 5 (explicit variable: PsyCap)		Equation 6 (explicit variable: creativity)	
	*B*	*t*	*B*	*t*	*B*	*t*
PsyCap					0.030	4.785^***^
Openness	0.257	9.792^***^	0.919	4.126^***^	0.229	4.904^***^
Conscientiousness	0.220	4.212^***^	0.872	3.506^***^	0.194	3.725^***^
Gender	0.084	2.289^*^	0.189	1.089	0.078	2.153^*^
Type of universities	0.245	7.258^***^	0.263	1.637	0.237	7.085^***^
Extraversion	0.096	2.627^**^	0.350	2.018^*^	0.085	2.357^*^
Neuroticism	−0.020	−0.838	−0.359	−3.119^**^	−0.009	−0.393
Agreeableness	−0.096	−2.240^*^	0.442	2.172^*^	−0.109	−2.571**
*R* ^2^	0.246		0.242		0.261	
*F*	50.776		49.818		48.181	

The results of the mediation analysis of model 3 are presented in Table 10. After controlling for type of university, gender and the other four dimensions of BFPT, we first found that conscientiousness positively predicted creativity, *B* = 0.220, *p* < 0.001 (Eq. 1). Second, conscientiousness positively predicted PsyCap, *B* = 0.872, *p* < 0.001 (Eq. 2). Third, after putting both independent and mediating variables into the equation, consciousness positively predicted creativity, *B* = 0.194, *p* < 0.001, PsyCap positively predicted creativity, *B* = 0.030, *p* < 0.001. The bias-corrected bootstrapping mediation test indicated that the process by which conscientiousness predicted creativity through PsyCap was significant, indirect effect = 0.026, 95% CI = (0.007, 0.049), which are presented in Table 9. The ratio of direct and mediated effects to the total effect was 88.18% and 11.82%, respectively, which was a partially mediated model. The mediated path diagram of model 3 is shown in Figure 2.

#### 4.3.4. Mediated influence pathways test of communication

In this section, the mediating paths of BFPT through PsyCap influencing communication were tested, yielding 2 significant mediating models: the model 4 was openness-PsyCap-communication, the model 5 was conscientiousness-PsyCap-communication. The results suggest that openness and conscientiousness both improve performance in communication partially by enhancing PsyCap.

The results of the mediation analysis of model 4 are presented in Table 11. After controlling for the type of universities, gender, and the other 4 dimensions of BFPT, we first found that openness positively predicted communication, *B* = 0.196, *p* < 0.001 (Eq. 1). Second, openness positively predicted PsyCap, *B* = 0.924, *p* < 0.001 (Eq. 2). Third, after integrating both independent and mediating variables into the equation, openness positively predicted communication, *B* = 0.171, *p* < 0.001, PsyCap positively predicted communication, *B* = 0.027, *p* < 0.001 (Eq. 3). The bias-corrected bootstrapping mediation test indicated that the process by which openness predicted communication through PsyCap was significant, indirect effect = 0.024, 95% CI = (0.009, 0.044), which are presented in Table 9. The ratio of direct and mediated effects to total effect was 87.76% and 12.24%, respectively, which was a partially mediated model. The mediated path diagram of model 4 is shown in Figure 2.

**Table 11:** Mediation model 4 test: openness-PsyCap-communication and model 5 test: conscientiousness-PsyCap-communication.

Predictors	Equation 7 (explicit variable: communication)		Equation 8 (explicit variable: PsyCap)		Equation 9 (explicit variable: communication)	
	*B*	*t*	*B*	*t*	*B*	*t*
PsyCap					0.027	4.667^***^
Openness	0.196	4.603^***^	0.924	4.127^***^	0.171	4.034^***^
Conscientiousness	0.192	4.053^***^	0.873	3.495^***^	0.169	3.579^***^
Gender	0.019	0.581	0.138	0.795	0.016	0.474
Type of universities	0.230	7.534^***^	0.285	1.772	0.223	7.345^***^
Extraversion	0.110	3.322^***^	0.356	2.044^*^	0.101	3.060^**^
Neuroticism	−0.038	−1.714	−0.358	−3.089^**^	−0.028	−1.290
Agreeableness	−0.071	−1.832	0.436	2.129^*^	−0.083	−2.144^*^
*R* ^2^	0.239		0.239		0.254	
*F*	49.627		49.465		46.980	

The results of the mediation analysis of model 5 are presented in Table 11. After controlling for the type of university, gender and the other 4 dimensions of BFPT, we first found that conscientiousness positively predicted communication, *B* = 0.192, *p* < 0.001 (Eq. 1). Second, conscientiousness positively predicted PsyCap, *B* = 0.873, *p* < 0.001 (Eq. 2). Third, after adding the independent and mediating variables into the equation, consciousness positively predicted communication, *B* = 0.169, *p* < 0.001, PsyCap positively predicted communication, *B* = 0.027, *p* < 0.001 (Eq. 3). The bias-corrected bootstrapping mediation test indicated that the process by which consciousness predicted communication through PsyCap was significant, indirect effect = 0.023, 95% CI = (0.007, 0.043), which are presented in the Table 9. The ratio of direct and mediated effects to total effect was 88.02% and 11.98%, respectively, which was a partially mediated model. The mediated path diagram of model 4 is shown in Figure 2.

#### 4.3.5. Mediated influence pathways test of collaboration

In this section, the mediating paths of BFPT through PsyCap influencing collaboration were tested, yielding 2 significant mediating models: the model 6 was extraversion-PsyCap-collaboration, the model 7 was openness-PsyCap-collaboration. The results suggest that extraversion and openness both improve performance in collaboration partially by enhancing PsyCap.

The results of the mediation analysis of model 6 are presented in Table 11. After controlling for type of university, gender and the other 4 dimensions of BFPT, we first found that extraversion positively predicted collaboration, *B* = 0.113, *p* < 0.001 (Eq. 1). Second, extraversion positively predicted PsyCap, *B* = 0.443, *p* < 0.05 (Eq. 2). Third, after putting both independent and mediating variables in the equation, extraversion positively predicted collaboration, *B* = 0.099, *p* < 0.001, PsyCap positively predicted collaboration, *B* = 0.032, *p* < 0.001 (Eq. 3). The bias-corrected bootstrapping mediation test indicated that the process by which extraversion predicted collaboration through PsyCap was significant, indirect effect = 0.014, 95% CI = (0.001, 0.028), which are presented in Table 9. The ratio of direct and mediated effects to the total effect was 87.61 and 12.39%, respectively, which was a partially mediated model. The mediated path diagram of model 6 is shown in Figure 2.

The results of the mediation analysis of model 7 are presented in Table 12. After controlling for the type of universities, gender, and the other 4 dimensions of BFPT, we first found that openness positively predicted collaboration, *B* = 0.111, *p* < 0.005 (Eq. 1). Second, openness positively predicted PsyCap, *B* = 0.800, *p* < 0.001 (Eq. 2). Third, after integrating both independent and mediating variables into the equation, openness positively predicted collaboration, *B* = 0.085, *p* < 0.05, PsyCap positively predicted collaboration, *B* = 0.032, *p* < 0.001 (Eq. 3). The bias-corrected bootstrapping mediation test indicated that the process by which openness predicted collaboration through PsyCap was significant, indirect effect = 0.014, 95% CI = (0.001, 0.028), which are presented in Table 9. The ratio of direct and mediated effects to the total effect was 87.61% and 12.39%, respectively, which was a partially mediated model. The mediated path diagram of model 2 is shown in Figure 2.

**Table 12:** Mediation model 6 test: extraversion-PsyCap-collaboration and model 7 test: openness-PsyCap-collaboration.

Predictors	Equation 10 (explicit variable: collaboration)		Equation 11 (explicit variable: PsyCap)		Equation 12 (explicit variable: collaboration)	
	*B*	*t*	*B*	*t*	*B*	*t*
PsyCap					0.032	6.648^***^
Extraversion	0.113	4.053^***^	0.443	2.552^*^	0.099	3.609^***^
Openness	0.111	3.076^**^	0.800	3.584^***^	0.085	2.401^*^
Gender	0.038	1.377	0.126	0.736	0.034	1.256
Type of universities	0.226	8.837^***^	0.258	1.625	0.217	8.673^***^
Conscientiousness	0.071	1.779	1,091	4.375^***^	0.037	0.925
Neuroticism	−0.014	−0.788	−0.329	−2.888^**^	−0.004	−0.222
Agreeableness	0.023	0.687	0.363	1.764	0.011	0.345
*R* ^2^	0.201		0.249		0.233	
*F*	39.319		51.745		41.291	

Taken together, these findings lead to the following BFPT-PsyCap-key competencies that mediate the effect pathways, as shown in Figure 2. Models 1–7 are partially mediated models. In summary, our findings provide an affirmation for Hypothesis 5.

## 5. Discussion

Based on previous relevant studies, this study was carried out on a random sample of university undergraduates in the form of a questionnaire, and statistical methods such as independent sample *t*-test, correlation analysis, and bootstrap-mediated effects analysis were used to systematically explore the relationship between the key competencies of BFPT, PsyCap, and college students, the following conclusions were obtained.

### 5.1. Strengthening the focus and cultivation of key competencies among college students

Key competencies influence all aspects of the college student career development lifecycle, including well-being (Austin et al., 2005), academic achievement (O’Boyle et al., 2011), and job performance (Petrides et al., 2004), and physical fitness (Martins et al., 2010). The results of the descriptive analysis showed that Chinese college students were generally at a high level, with the highest to lowest scores being communication, collaboration, critical thinking and creativity. Compared to communication and collaboration, there is still room for improvement in critical thinking and creativity. The Chinese higher education system, with its excessive emphasis on examination-oriented education, can be considered as one of the factors that inhibits critical thinking (Baser et al., 2016) and inhibits creativity abilities (Ke and Liang, 2023). Critical thinking education is a topic that is currently receiving a lot of attention in university education worldwide, and creativity education is a topic that is receiving even more attention in countries that are focused on innovation-driven development. To meet the high-quality development requirements of China, the education system needs to focus more on nurturing the critical thinking and creativity of college students.

The results also showed that there was no significant difference in the key competencies of college students in terms of gender (Hyde, 2005), while there were significant differences in the key competencies of critical thinking and creativity of college students in terms of the type of university. Research has shown that both boys and girls have the same potential to develop their key competencies, and the results can challenge past gender stereotypes and encourage a more open attitude towards the abilities and potential of both men and women. Since gender does not have a significant effect on key competencies in college students, personality traits may be a more accurate predictor of individual behavior and competence than gender (Barrick et al., 2001). This may lead researchers to further explore the relationship between personality and competence. The differences in key competencies revealed by university type indicate that key universities may prioritize nurturing critical and innovative thinking in students, while general institutions might focus more on the transmission of knowledge and skill training. Furthermore, the results could be related to social and employment pressures, as these traits are considered key factors in a competitive job market.

### 5.2. Utilizing inherent personality traits as catalysts for individual development

Pearson’s correlation analysis was used to study the relationship between BFPT, PsyCap, and key competencies of college students; the results showed that there were significant correlations between the three. Significant correlations suggested that PsyCap may be a crucial contributing factor to the development of key competencies.

According to the study findings, except for neuroticism, which was a significant negative predictor of the key competencies of college students, the other dimensions of extraversion, openness, agreeableness, and conscientiousness were all significant positive predictors of the key competences, highlighting the importance of mental health support in educational settings (Kutcher et al., 2016). For example, in alignment with extant literature which identifies academic specific anxiety as a significant negative predictor of performance (Credé and Kuncel, 2008), the present investigation further revealed that students who tend to experience emotional instability or negative emotions such as anxiety and depression may face more challenges in developing key competencies. Educators could design different strategies or resources for students with different personality traits, aiming to minimize the potential drawbacks of traits such as neuroticism and maximize the benefits of traits such as conscientiousness, extraversion, agreeableness, and openness. In summary, these results can pave the way for more research into personalized education strategies based on personality traits.

### 5.3. Unveiling the interwoven pathways of personality, Psycap, and key competencies

A regression analysis was conducted to investigate the influence of BFPT, PsyCap, and key competencies among college students. The specific effects between the three variables were examined by mediation. According to research findings, PsyCap played a partially mediating influence effect between BFPT and key competencies. Therefore, strengthening the development of PsyCap is of positive significance for the enhancement of key competencies of college students.

More specifically, the mediating effects revealed that PsyCap served as a partial mediator in the relationships between neuroticism and critical thinking, openness and creativity, conscientiousness and creativity, openness and communication, conscientiousness and communication, extraversion and collaboration, as well as openness and collaboration.

As for why the mediating effect of PsyCap between BFPT and key competencies exists? We believe that the relationship between these three can be understood in greater depth in conjunction with the conservation of resources theory (COR). Hobfoll (1989) has asserted that individuals seek to acquire, maintain, and protect the resources that are valuable to them, and that both the loss and acquisition of these resources have important psychological and behavioral effects on individuals. Specifically, the Big Five’s contribution to PsyCap can be described on the basis of COR: openness in individuals often leads to a willingness to explore new knowledge and experiences, facilitating the acquisition of new skills. Conscientiousness, with its heightened sense of responsibility, can pave the way for superior resource management and organizational skills. Extraversion often correlates with resourcefulness in social interactions, enabling such individuals to gain more social support. Agreeableness tends to enhance one’s ability to foster positive relationships, increasing their social resources. Conversely, while high neuroticism suggests lower emotional stability, those with low neuroticism typically have better coping strategies and demonstrate resilience. BFPT can be viewed as initial resources or mediators of resource acquisition, which directly and indirectly influence an individual’s PsyCap. PsyCap, as a comprehensive psychological resource, promotes the development of key competencies. Individuals with high PsyCap are more likely to adopt positive strategies to cope with challenges and are more willing to learn and adapt, thus accumulating and enhancing their core competencies. This process is closely linked to the resource gain spiral effect (Hobfoll, 2002) in COR theory, in which the initial acquisition of resources facilitates the accumulation of further resources.

On the other hand, in conjunction with the life-cycle approach (LCA) (Roberts et al., 2006), personality traits may also be modifiable. BFPT are fundamentally innate and manifest early in life. They inherently influence an individual’s PsyCap and competency development, providing a foundational framework from which individual behaviors and capacities emerge. LCA posit that, contrary to previous assumptions, personality traits are not rigid. Throughout the lifecycle, individuals can actively work to mitigate negative traits and accentuate positive ones. This dynamic nature of personality implies adaptability and the potential for growth at various life stages. Recognizing the potential for personality evolution, one can deduce a mediated pathway where alterations in BFPT, whether naturally occurring or through intentional interventions, can impact PsyCap. This, in turn, influences the growth and refinement of competencies. In essence, as personality evolves, so does the nature and magnitude of its influence on psychological resources and capabilities.

The data suggested a significant influence of PsyCap as a mediator between BFPT and key competencies, with varying degrees of mediation effects. In particular, the highest mediation effect was found in the relationship between openness and collaboration (22.72%), indicating that PsyCap plays a substantial role in these areas. Furthermore, both openness and conscientiousness demonstrated considerable mediation effects on creativity, which implied meaningful interactions between these traits and PsyCap. In contrast, neuroticism presented a lower mediation effect (5.97%) on critical thinking, which may underscore the lesser influence of neuroticism or the mitigating role of PsyCap. In conclusion, enhancing PsyCap, tailored to specific personality traits, could be a strategic approach to fostering key competencies in students. Within the existing body of research, investigators have discovered that PsyCap and BFPT serve as individual predictors of the development of key competencies in university students (Luthans et al., 2007; Poropat, 2009). However, our study takes this understanding a step further. We found that PsyCap not only predicts competency development but also performs a partial mediating role between BFPT and key competencies, effectively bridging the two. This offers implications for the development of personalized education interventions.

## 6. Limitations and prospects

Due to the limitations of the research method and sample collection, the following shortcomings still exist. In terms of collecting research data, only undergraduate students were selected as the research sample, and variables such as their grade and major were not collected, which limits the generalizability of the findings. Therefore, in future studies, samples with richer characteristics can be selected to make the findings more generalizable. Furthermore, this article adopts a cross-sectional approach, while key competencies and PsyCap are a continuous process of change, so a longitudinal approach can be considered in future research to explore the factors influencing key competencies of college students in greater depth.

## Data availability statement

The raw data supporting the conclusions of this article will be made available by the authors, without undue reservation.

## Ethics statement

The studies involving humans were approved by The Ethics Committee at the Beijing Forestry University. The studies were conducted in accordance with the local legislation and institutional requirements. Written informed consent for participation in this study was provided by the participants’ legal guardians/next of kin.

## Author contributions

AH: writing-original draft, formal analysis, methodology, investigation; XL: validation, visualization, software, data analysis; HS: led the idea for this article and worked on writing-review & editing, funding acquisition, supervision, project administration. All authors contributed to the article and approved the submitted version.

## Funding

This study was supported by Research on Xi Jinping’s Discourse on Talent Work, a project of the Xi Jinping Research Project on Socialist Thought with Chinese Characteristics for a New Era. [# 22XNQ010]. Thanks to the support of this fund.

## Conflict of interest

The authors declare that the research was conducted in the absence of any commercial or financial relationships that could be construed as a potential conflict of interest.

## Publisher’s note

All claims expressed in this article are solely those of the authors and do not necessarily represent those of their affiliated organizations, or those of the publisher, the editors and the reviewers. Any product that may be evaluated in this article, or claim that may be made by its manufacturer, is not guaranteed or endorsed by the publisher.

## Footnotes

^1^Refers to Chinese universities in the list of Project 211 and Project 985.

^2^Refers to Chinese universities outside the list of Project 211 and Project 985.
